# NDRG2 phosphorylation provides negative feedback for SGK1-dependent regulation of a kainate receptor in astrocytes

**DOI:** 10.3389/fncel.2015.00387

**Published:** 2015-10-06

**Authors:** Veronika Matschke, Carsten Theiss, Michael Hollmann, Eric Schulze-Bahr, Florian Lang, Guiscard Seebohm, Nathalie Strutz-Seebohm

**Affiliations:** ^1^Department of Cardiovascular Medicine, Institute for Genetics of Heart Diseases (IfGH), University Hospital MuensterMuenster, Germany; ^2^Department of Cytology, Institute of Anatomy, Ruhr University BochumBochum, Germany; ^3^Department of Biochemistry I - Receptor Biochemistry, Ruhr University BochumBochum, Germany; ^4^Department of Physiology, University TuebingenTuebingen, Germany

**Keywords:** glutamate receptor, GluR6, trafficking, phosphorylation, regulation, fluorescence, immuno-cytochemistry, line plot

## Abstract

Glutamate receptors play an important role in the function of astrocytes. Among their tasks is the regulation of gliotransmission, gene expression and exocytosis of the tissue-type plasminogen activator (tPA), which has an enhancing effect on N-methyl-D-aspartate (NMDA) receptors and thus prevent over-excitation of neighboring neurons. The kainate receptor GluK2, which is expressed in neurons and astrocytes, is under tight regulation of the PI3-kinase SGK pathway as shown in neurons. SGK1 targets include N-myc downstream-regulated genes (NDRGs) 1 and 2 (NDRG1, NDRG2), proteins with elusive function. In the present study, we analyzed the effects of SGK1, NDRG1, and NDRG2 on GluK2 current amplitude and plasma membrane localization in astrocytes and heterologous expression. We demonstrate that NDRG1 and NDRG2 themselves have no effect on GluK2 current amplitudes in heterologous expressed ion channels. However, when NDRG2 is coexpressed with GluK2 and SGK1, the stimulating effect of SGK1 on GluK2 is suppressed both in heterologous expression and in astrocytes. Here, we reveal a new negative feedback mechanism, whereby GluK2 stimulation by SGK1 is regulated by parallel phosphorylation of NDRG2. This regulation of GluK2 by SGK1 and NDRG2 in astrocytes may play an important role in gliotransmission, modulation of gene expression and regulation of exocytosis of tPA.

## Introduction

Ionotropic glutamate receptors play an important role in the function of astrocytes, the most abundant glial cells in the brain (Cornell-Bell et al., 1990; Kim et al., 1994; Casse et al., 2012). These receptors are classified into three types and are named according to their selective agonists N-methyl-D-aspartate (NMDA), γ-amino-3-hydroxy-5-methyl-4-isoxazole propionic acid (AMPA), and kainate.

Expression of kainate receptor subunits (GluK) was examined in various animal models, and various brain regions in astrocytes (García-Barcina and Matute, 1996; Paschen et al., 1996; Brand-Schieber et al., 2004). Kainate depolarizes primary astrocytes from the brain of rats and moreover, increases the intracellular calcium concentration in astrocytes of hippocampal slices (Cornell-Bell et al., 1990; McNaughton and Hunt, 1992; Kim et al., 1994). Since kainate receptors are partially Ca^2+^-permeable (Paschen et al., 1996; Lowe et al., 1997; Bernard et al., 1999), important regulatory mechanisms can be activated through activation of GluK receptors and the resulting influx of Ca^2+^ ions. The increase in intracellular Ca^2+^ after activation of GluK receptors by kainate (Cornell-Bell et al., 1990; Kim et al., 1994; Liu et al., 2004) triggers Ca^2+^ waves within the astrocytic syncytium and may lead to release of gliotransmitters, e.g., glutamate or the tissue-type plasminogen activator (tPA) from astrocytes (Liu et al., 2004; Mothet et al., 2005; Casse et al., 2012). The released glutamate has further effects on neighboring hippocampal neurons by triggering a slow, transient current, which in turn can activate NMDA receptors (Angulo et al., 2004; Fellin et al., 2004) or by activating GluK5-containing kainate receptors on adjacent interneurons and thus increases their inhibitory postsynaptic currents (Rodríguez-Moreno and Lerma, 1998; Liu et al., 2004). Thus, kainate receptors in astrocytes serve as sensors for extracellular glutamate in astrocytes thereby preventing excitotoxicity.

Due to their key functions glutamate receptors like NMDA receptors or kainate receptors are subject to very tight regulation. The GluK2 subunit is functionally modulated by the serum- and glucocorticoid-inducible kinase 1. Expression of SGK1 in the human brain has been shown both in neurons and in oligodendrocytes (Webster et al., 1993; Imaizumi et al., 1994; Wärntges et al., 2002). The expression in astrocytes is currently debated, as there are insufficient, valid direct protein observations for these cells (Wärntges et al., 2002; Stichel et al., 2005; Slezak et al., 2013; Wu et al., 2013). SGK1 transcription is stimulated by a wide variety of hormones including glucocorticoids and mineralocorticoids as well as by cell stress, such as cell shrinkage, increase of cytosolic Ca^2+^ activity and by cellular energy depletion (Webster et al., 1993; Brennan and Fuller, 2000; Ullrich et al., 2005). SGK1 phosphorylates target proteins at the classical SGK1 consensus sequence R-X-R-X-X-(S/T)-h, with X being a random amino acid, R an arginine, S/T serine or threonine and an hydrophobic amino acid (Kobayashi and Cohen, 1999). As shown by electrophysiological techniques and immunohistochemical studies on *Xenopus laevis* oocytes and hippocampal neurons SGK1 increases GluK2 cell membrane protein abundance and thus glutamate-induced current amplitude (Strutz-Seebohm et al., 2005). The disclosed modulation of GluK2 activity is due to indirect phosphorylation of the iGluR via the activation of a hitherto elusive signaling cascade that results in an increase of the plasma membrane expression of the iGluR. However, there are also proteins that are directly phosphorylated by SGK1. These proteins include two representatives of the N-myc downstream-regulated gene (NDRG) protein family, NDRG1 and NDRG2 (Murray et al., 2004; Okuda et al., 2008).

The NDRG protein family consists of four members (NDRG1-4). All NDRG proteins were detected in the central nervous system. NDRG3 and NDRG4 are ubiquitously expressed, whereas NDRG1 and NDRG2 are only detected in oligodendrocytes and astrocytes (Okuda et al., 2008). NDRG1 has five phosphorylation sites for SGK1 (T328, S330, T346, and T356, T366). NDRG2 has three phosphorylation sites for SGK1, which are equivalent to the first three sites in NDRG1 (T330, S332, and T348; Murray et al., 2004).

NDRG2 is expressed in many parts of the brain, including the hippocampus (Nichols, 2003; Shen et al., 2008). The protein expression in adult animals is limited to astrocytes in these brain areas (Nichols, 2003; Okuda et al., 2008; Flügge et al., 2014). Like SGK1, NDRG2 is modulated by glucocorticoids at the transcriptional level. Exposure to dexamethasone (Dexa) leads to increased NDRG2 mRNA in astrocytes (Nichols, 2003; Liu et al., 2011; Takahashi et al., 2011). Currently, any knowledge of the precise function of NDRG2 in astrocytes is limited. It was suggested that NDRG2 may be important for neurogenesis (Nichols, 2003; Shen et al., 2008; Liu et al., 2011), cell proliferation and differentiation within murine brains (Shen et al., 2008; Liu et al., 2011; Takeichi et al., 2011). NDRG2 has also distinct effects on ion channels (Wielpütz et al., 2007; Li et al., 2011). Furthermore, NDRG2 modulates the half-life of the Na^+^/K^+^-ATPase (Li et al., 2011).

The aim of this study was to gain insight into the regulatory mechanism how the proteins NDRG1 and NDRG2, which are directly phosphorylated by SGK1, influence the SGK1-activated signaling cascade that ultimately leads to an increase in membrane expression of GluK2.

## Materials and Methods

### cDNAs and Mutagenesis of Vector Constructs

The following cDNAs were used: GluK2/pSGEM, GluK2/pSGEM-enhanced green fluorescent protein (EGFP), SGK1/pGHJ, NDRG1/pCMV-Sport6, NDRG2/pCMV-Sport6. The point mutations were introduced into mouse NDRG2/pCMV-Sport6 by using the QuickChange Lightning Site-Directed Mutagenesis Kit (Agilent Technologies, Santa Clara, CA, USA). Success of mutagenesis was verified by sequencing.

### Synthesis of cRNA

Template DNA was linearized with an appropriate restriction enzyme. cRNA was synthesized from 1 μg linearized DNA using an *in vitro* transcription kit (mMessage mMachine SP6 or T7 kit—Ambion, Life Technologies, Darmstadt, Germany). Concentrations of cRNA were examined by photospectrometry (NanoDrop ND-100) and the quality of the transcript was verified by agarose gel electrophoresis.

### Heterologous Expression in *Xenopus laevis* Oocytes

cRNA (GluK2 4 ng, SGK1 6 ng, NDRG2 6 ng) was injected into stage V–VI *Xenopus* oocytes, provided by Ecocyte Bioscience (Castrop-Rauxel, Germany). All measurements were performed 5–6 days after injection of cRNA. Oocytes were incubated at 17–18°C.

### Electrophysiological Recordings

The two-electrode voltage clamp technique (Goldin, 2006) was used to record whole cell currents in *Xenopus* oocytes at room temperature (22–23°C). Data were acquired and analyzed using pCLAMP 8.0 software (Axon Instruments, Sunnyvale, CA, USA). Data analyses were done with pClamp/Clampex software 8.0 (Axon Inc., CA, USA) and Prism 6.1 software (GraphPad Software, San Diego, CA, USA). For voltage clamp experiments, agonist solutions were prepared in ND-96 buffer (mM: NaCl 96, CaCl_2_ 1.8, KCl 2.0, MgCl_2_ 1.0 and HEPES–NaOH 5, pH 7.2 with NaOH). Current and voltage electrodes were filled with 3 M KCl and had resistances of 0.5–1.2 MΩ. The oocytes were held at −80 mV and agonist (300 μM glutamate; Sigma-Aldrich, Schnelldorf, Germany) was superfused for 20 s at a flow rate of 10–14 ml min^−1^. Prior to agonist application, the oocytes were incubated for 8 min in concanavalin A (ConA) to prevent GluK2 desensitization. Data are provided as means ± SEM, *n* represents the number of oocytes investigated. All experiments were repeated with at least three batches of oocytes; in all repetitions qualitatively similar data were obtained. Data were tested for significance using Student’s *t*-test and ANOVA, and results with *p* < 0.05 were considered statistically significant.

### Preparation of Cell Surface Proteins and Cytosolic Proteins out of *Xenopus* Oocytes

To identify the fraction of receptor protein inserted in the plasma membrane, surface proteins were tagged with biotinylated ConA and isolated by streptavidin-sepharose-mediated precipitation of the biotinyl-ConA-protein complex as previously described by Strutz-Seebohm et al. (2003). For determination of cytosolic proteins, intact oocytes were homogenized with a teflon pestle in H-buffer (25 μl/oocyte; 100 mM NaCl, 20 mM Tris-HCl, pH 7.4, 1% Triton X-100, 1 mM phenylmethylsulfonyl fluoride plus a mixture of proteinase inhibitors; Complete^TM^ tablets, Roche Applied Science, Penzberg, Germany). After centrifugation for 2 min at 16,000×g, the supernatants were supplemented with 9 μl of SDS-PAGE loading buffer (0.8 M ß-mercaptoethanol, 6% SDS, 20% glycerol, 25 mM Tris-HCl, pH 6.8, 0.1% bromphenol blue).

### Gel Electrophoresis and Western Blotting

Proteins from homogenized oocytes were separated by SDS gel electrophoresis and transferred to nitrocellulose membranes. Blots were blocked in 1× PBS containing 1% RotiBlock (Roth, Karlsruhe, Germany) for at least 2 h at room temperature. For the detection of GluK2, primary goat polyclonal IgG anti-GluK2 antibody (1 μg μl^−1^) and secondary horseradish peroxidase-conjugated rabbit anti-goat antibody (1:1000 dilution) were used. For verification of protein levels, Ponceau Red staining was performed. For the detection of NDRG2, primary goat polyclonal IgG anti-NDRG2 antibody (1 μg μl^−1^) and secondary horseradish-peroxidase-conjugated rabbit anti-goat antibody (1:1000 dilution) were used. For verification of protein levels, Ponceau Red staining was performed.

### Confocal Microscopy of Oocytes

C-terminal EGFP-tagged ion channels expressed in *Xenopus* oocytes were imaged using a TCS SP2 AOBS confocal microscope (Leica Microsystems, Wetzlar, Germany). EGFP was excited at 488 nm, and fluorescence was detected between 500 and 600 nm in combination with 20× objective (HC PL APO Ibd.BL 20×/0.7 water). The data were analyzed with ImageJ (U.S. National Institutes of Health, Bethesda, MD, USA).

### Primary Hippocampal Cell Culture

The studies have been performed under the terms of the German animal protection law. Newborn wild type Wistar rats of postnatal day 0–2 (P0–P2) were used for cultivation of primary rat hippocampal astrocytes. Primary hippocampal astrocytes were prepared according to a similar protocol as described by Seebohm et al. (2012). In brief, the hippocampi of P0–P2 new-born Wistar rats were minced in ice cold MPBS^+/+^ (modified phosphate-buffered saline supplemented with 0.25 mM CaCl_2_, 5.8 mM MgCl_2_, 10 mM HEPES, 1 mM sodium pyruvate, 6 μg/ml DNaseI, 1 mg/mL bovine serum albumin (BSA), 10 mM glucose, 25 U/mL penicillin, 25 μg/ml, streptomycin, 2 mM glutamine, 5 mg/mL phenol red, 4 mM NaOH) and digested with 10% trypsin in MPBS^−/−^ (MPBS^+/+^ without CaCl_2_ and MgCl_2_) for 7 min while shaking at 37°C and 750 rpm. After removing of the remaining tissue, the supernatant (containing dissociated cells) was diluted two-fold with RPMI/10% fetal calf serum [FCS; 2 mM glutamine, 10% FCS, 25 U/mL penicillin, 25 μg/ml, streptomycin, 0.00375% insulin, 5 mM glucose, 10 mM HEPES, in RPMI 1640 (PAA)] to terminate digestion. Residual tissue pieces were dissociated by trituration in MPBS^−/−^ with pipette tips three times. The cells were collected by centrifuging at 500×g for 10 min. The cells were dissociated in RPMI/10% FCS, seeded at a density of 125,000 cells per 12 mm collagen-coated glass cover slip and cultivated at 37°C and 5% CO_2_. For preparation of astrocyte enriched cultures, the medium was retrieved after 1.5 h. The following day, the media was exchanged against neurobasal medium (Invitrogen, Darmstadt, Germany) containing B-27 serum-free supplement (Invitrogen; 1× B27, 2 mM glutamine, 100 U/ml penicillin, 100 μg/ml streptomycin in neurobasal medium). Every second to third day the media was exchanged against fresh pre-incubated medium. On day three and five of cultivation, transfection of control siRNA (sequence: UAGCGACUAAACACAUCAA; Thermo Scientific, Schwerte, Germany) or a siRNA pool against NDRG2 (ACCCAAACGUCCAGCGAUA; CGUUGAAGGUCUUGUUCUC; UUUCAAGUACUUCGUGCAA; GCAUACAUUCUGUCACGAU; Thermo Scientific, Schwerte, Germany) was performed with Effectene (Qiagen, Hilden, Germany). The cells were used for experiments after 9 days *in vitro*. The media was removed and fresh neurobasal medium without B-27 was added to the cells 4 h prior to stimulations of SGK1 with dexamethasone (1 μM; Sigma-Aldrich, Schnelldorf, Germany). Finally, the cells were fixed with 4% paraformaldehyde in PBS or harvested for preparation of intracellular proteins.

### Preparation of Intracellular Proteins Out of Astrocyte Enriched Cultures

To determine expression levels of SGK1 and NDRG2, astrocyte enriched cultures were prepared and treated with siRNA and/or Dexa as described above. Control cells were treated with Effectene alone. For lysis of hippocampal cells, the 3.5-cm dish plate was washed with 1× PBS to remove residual media. Then, 500 μl of ice-cold 1× PBS/3.5 cm dish was added and the cells were mechanically released, followed by centrifugation at 4°C for 10 min at 14,000×g. 20 μl of 1× cell lysis buffer (Cell Signaling Technology, Danvers, MA, USA) was added and the cells incubated on ice for 5 min. After centrifugation at 4°C for 10 min at 14,000×g, the supernatant was removed and stored at −80°C until needed. Prior to gel electrophoresis and Western blotting, a protein assay (DC Protein Assay, Bio-Rad Laboratories, Munich, Germany) was performed to determine the total protein concentrations. For Western blotting, 2–5 μg of total protein was applied per lane. For the detection of NDRG2, primary goat polyclonal IgG anti-NDRG2 antibody (1 μg μl^−1^) and secondary horseradish-peroxidase-conjugated rabbit anti-goat antibody (1:1000 dilution) were used. For the detection of SGK1, primary mouse monoclonal IgG_2b_ anti-SGK1 antibody (1 μg μl^−1^) and secondary horseradish-peroxidase-conjugated sheep anti-mouse antibody (1:1000 dilution) were used. The specificity of primary antibodies was tested by Western blot by using blocking peptides for NDRG2 (#sc-19468 P, Santa Cruz, Heidelberg, Germany) and SGK (sc-28338 P, Santa Cruz, Heidelberg, Germany) according to manufactures protocols. For verification of protein levels, Ponceau Red staining was performed as well as detection of calnexin (primary antibody: 1 μg μl^−1^; secondary antibody: 1:1000 dilution).

### Immunocytochemistry

For immunocytochemistry, mixed hippocampal cultures were fixed with 4% paraformaldehyde. The primary cells were incubated with 0.3% Triton X-100 in PBS for 10 min. Thereafter, cells were washed three times with PBS and unspecific binding sites were blocked with 5% goat-serum in PBS for 30 min. Further the cells were incubated with primary antibodies overnight at 4°C (rabbit anti-glial fibrillary acidic protein (GFAP); goat anti-GluK2; mouse anti-SGK1; goat anti-NDRG2). The samples were then reacted with secondary antibodies for 2 h at room temperature (anti-rabbit AlexaFluor594; anti-mouse AlexaFluor488; anti-goat AlexaFluor488). Nuclei were stained with DAPI (1 μg/ml; #D9542, Sigma-Aldrich, Schnelldorf, Germany). Finally, cultures were rinsed in PBS and coverslipped in fluoromount (#F4680, Sigma-Aldrich, Schnelldorf, Germany). Samples were imaged using an inverted Zeiss Cell Observer fluorescence microscope equipped with the respective filter sets, an ApoTome and an Axiocam MRm in combination with Zeiss 63× oil immersion lens (Plan-Apochromat, 63×/1.4 Oil DICII, Zeiss, Oberkochen, Germany). Overlay and analysis were performed with AxioVision software (Zeiss, Oberkochen, Germany).

Hippocampal mixed and astrocyte enriched cultures were stained with GFAP or Neurofilament N specific for astrocytes and neurons, respectively to provide an overview of culture composition (Supplementary Figure S1).

### Relative Abundance of GluK2 in Astrocytes Determined by Fluorescence Microscopy

The images were analyzed using AxioVision (Zeiss, Oberkochen, Germany) and Prism software (GraphPad Software, San Diego, CA, USA). After scanning an astrocyte using an oil immersion objective (63×), a fluorescence profile omitting the nucleus within the cell soma was created. The fluorescence intensities within such a profile were normalized to the highest value. The increase of the fluorescence intensity within the first micrometer of the cell was described by a linear equation, and the regression coefficient was determined. To investigate changes in the localization of the glutamate receptor at the astrocyte membrane, the determined regression coefficients were compared under different conditions. If the GluK2 fluorescence increases rapidly within the first micrometer of the cell, i.e., at the membrane, the regression coefficient is correspondingly larger, indicating an increase in the number of receptors in close vicinity to the astrocyte membrane.

### Antibodies Used for Western Blotting and Immunocytochemistry

The following primary antibodies were used for Western blotting and immunostaining:

**Table:** 

goat anti-GluK2	#sc-7618	Santa Cruz Heidelberg, Germany	goat anti-NDRG2	#sc-19468	Santa Cruz Heidelberg, Germany	mouse anti-SGK1	#sc-28338	Santa Cruz Heidelberg, Germany	rabbit anti-Calnexin	#sc-11397	Santa Cruz Heidelberg, Germany	rabbit anti-GFAP	#G9269	Sigma-Aldrich, Schnelldorf, Germany	rabbit anti-MAP2	#M3696	Sigma-Aldrich, Schnelldorf, Germany

The following secondary antibodies were used for Western blotting:

**Table:** 

HRP-conjugated rabbit anti-goat	#A5420	Sigma-Aldrich, Schnelldorf, Germany	HRP-conjugated donkey anti-rabbit	#NA934	GE Healthcare, Solingen, Germany	HRP-conjugated sheep anti-mouse	#NA931	GE Healthcare, Solingen, Germany

The following secondary antibodies were used for immunocytochemistry:

**Table:** 

AlexaFluor594 goat anti-rabbit	#A11012	Life Technologies, Darmstadt, Germany	AlexaFluor488 donkey anti-mouse	#A21202	Life Technologies, Darmstadt, Germany	AlexaFluor488 rabbit anti-goat	#A11078	Life Technologies, Darmstadt, Germany

## Results

### NDRG2 Suppressed the GluK2 Current-Stimulating Effect of SGK1 in a Concentration-Dependent Manner

**Figure 1:**
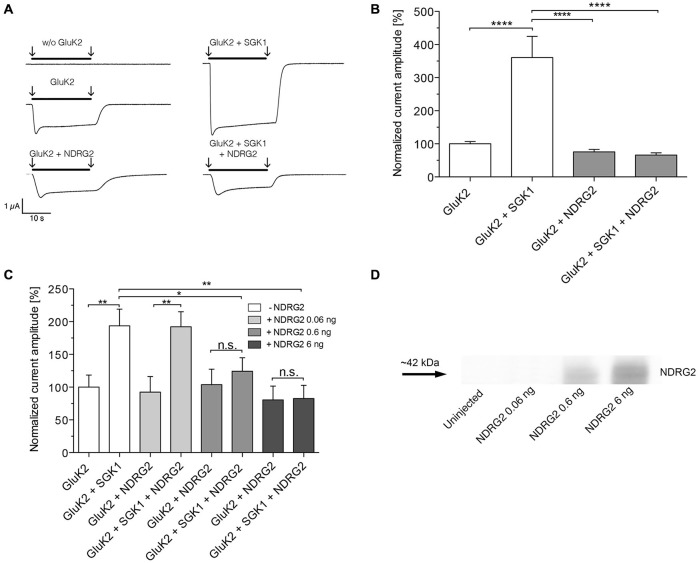
**Suppression of the GluK2-stimulating effect of SGK1 by NDRG2. (A)** Representative current traces measured in *Xenopus* oocytes in response to super fusion with 300 μM glutamate. Black bars above the current traces indicate the application of glutamate (arrows indicate start and stop of application). All currents were measured at −80 mV and after pretreatment of oocytes with ConA to minimize desensitization. **(B)** GluK2 current amplitudes in oocytes expressing GluK2 (*n* = 106), GluK2 + SGK1 (*n* = 85), GluK2 + NDRG2 (*n* = 73) and GluK2 + SGK1 + NDRG2 (*n* = 69) were measured and are shown normalized to GluK2 currents. Significant differences are indicated by ****p < 0.0001. **(C)** GluK2 current amplitudes in oocytes expressing GluK2 (*n* = 15), GluK2 + SGK1 (*n* = 11), GluK2 + NDRG2, and GluK2 + SGK1 + NDRG2 at different amounts of NDRG2 (0.06, 0.6 and 6 ng) and same amount of SGK1 (6 ng) were measured and are shown normalized to GluK2 currents (*n* = 11–14). Significant differences are indicated by **p < 0.01, *p < 0.05, n.s. = not significant. **(D)** Exemplary Western blot of cytosolic expressed NDRG2 after injection of the corresponding amount of cRNAs and an expression time of 6 days. Oocytes were homogenized. Samples including controls from uninjected oocytes were separated on an SDS gel, blotted onto a nitrocellulose membrane and probed with an anti-NDRG2 antibody. The NDRG2 protein has an apparent molecular mass of ~42 kDa.

The present study explored the modulatory effect of NDRG2 on the SGK1-activated signaling cascade, which leads to a change in membrane expression of GluK2. For this purpose, the channel was heterologously expressed in *Xenopus laevis* oocytes, with or without SGK1 and/or NDRG2. Glutamate-induced current amplitudes were studied by two-electrode voltage clamp measurements. As shown in Figure 1 application of glutamate induced a strong current in *Xenopus* oocytes overexpressing GluK2. Expression of SGK1 in addition to GluK2 significantly increased the GluK2 current. By contrast, coexpression of GluK2 with NDRG2 did not alter the glutamate-induced current amplitude compared with GluK2 alone. Thus, the stimulating effect of SGK1 on GluK2 was not detectable when GluK2 was coexpressed with SGK1 and NDRG2 (Figures 1A,B). As illustrated in Figure 1C, the suppressive effect of NDRG2 was dependent on the amount of NDRG2 cRNA injected and statistically significant at 0.6 and 6 ng NDRG2, but not at 0.06 ng. The expression of NDRG2 proteins after injection of different amounts of cRNA was detected by Western blot (Figure 1D). A clear protein band can be observed by usage of 0.6 and 6 ng NDRG2 cRNA, whereas no expression of NDRG2 can be observed at injection of 0.06 ng NDRG2 cRNA.

### Suppressive Effect of NDRG2 on SGK1-Dependent Modulation of GluK2 Membrane Expression

**Figure 2:**
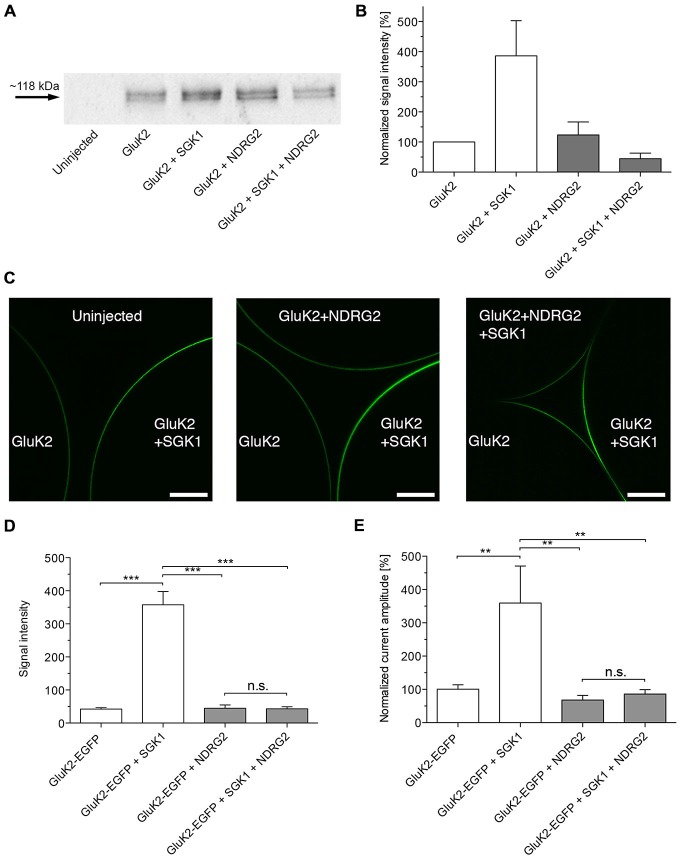
**Suppressive effect of NDRG2 on SGK1-dependent modulation of GluK2 membrane expression. (A)** Representative Western blot demonstrating the suppressive effect of NDRG2 on SGK1-dependent modulation of GluK2 membrane expression. Glycosylated plasma membrane proteins expressed in oocytes were labeled with biotinylated concanavalin A (ConA). Oocytes were homogenized and plasma membrane proteins were streptavidin-precipitated. Samples including controls from uninjected oocytes were separated on an SDS gel, blotted, onto a nitrocellulose membrane and probed with an anti-GluK2 antibody. The double band might represent different glycosylation forms of the GluK2 protein (Strutz-Seebohm et al., 2006). The GluK2 protein has an apparent molecular mass of ~118 kDa. **(B)** Bar graph showing relative abundance of GluK2 plasma membrane protein (*n* = 4). The band intensity was quantified by arithmetic analysis using the software ImageJ. **(C)** Imaging of enhanced green fluorescent protein (EGFP)-GluK2 expression in *Xenopus* oocytes at different conditions of proteins expressed. Confocal laser-scanning microscopy of control oocytes and oocytes expressing GluK2-EGFP, GluK2-EGFP + SGK1, GluK2-EGFP + NDRG2 or GluK2-EGFP + SGK1 + NDRG2. Representative images are presented in which the optical slice approximately bisects the oocyte. Scale bars (white) indicate 150 μm. **(D)** Bar graph showing normalized (to GluK2-EGFP alone) mean signal intensities of GluK2-EGFP in oocytes at different constellations of injected cRNA (*n* = 11–51). The intensity was quantified by arithmetic analysis using the software ImageJ. Significant differences are indicated by ***p < 0.0001, n.s. = not significant. **(E)** GluK2-EGFP current amplitudes in oocytes expressing GluK2-EGFP (*n* = 17), GluK2-EGFP + SGK1 (*n* = 13), GluK2-EGFP + NDRG2 (*n* = 14), and GluK2-EGFP + SGK1 + NDRG2 (*n* = 12) were measured and are shown normalized to GluK2-EGFP currents. All currents were measured at −80 mV and after pretreatment of oocytes with ConA to minimize desensitization. Significant differences from current amplitudes in oocytes are indicated by **p < 0.01, n.s. = not significant.

The increase of GluK2 currents following coexpression of SGK1 resulted from an enhanced abundance in the plasma membrane (Strutz-Seebohm et al., 2005). Thus, chemiluminescence and a C-terminal EGFP-tagged GluK2 were used to quantify GluK2 protein abundance in the cell membrane of *Xenopus laevis* oocytes expressing GluK2 with or without SGK1 and/or NDRG2. As shown in Figures 2A,B, the protein abundance of GluK2 was significantly enhanced in *Xenopus* oocytes expressing GluK2 together with SGK1 compared with GluK2 alone. No significant effect on GluK2 protein abundance was observed following coexpression of NDRG2. The stimulating effect of SGK1 on GluK2 membrane expression was also not detectable when GluK2 was coexpressed with SGK1 and NDRG2. The same observations were made with the EGFP-tagged GluK2 protein (Figures 2C,D). Coexpression of GluK2-EGFP with SGK1 increased membrane fluorescence in GluK2-EGFP expressing oocytes significantly. Expression of GluK2-EGFP in addition to NDRG2 showed no effect on GluK2-EGFP protein abundance. The stimulating effect of SGK1 on GluK2-EGFP membrane expression was again abrogated following additional coexpression of NDRG2. Properties of currents expressed in oocytes and modulatory effects of the proteins SGK1 and NDRG2 were not affected by the C-terminal EGFP tag (Figure 2E).

### NDRG1 did not Modulate SGK1-Dependent Stimulation of GluK2 Membrane Expression

**Figure 3:**
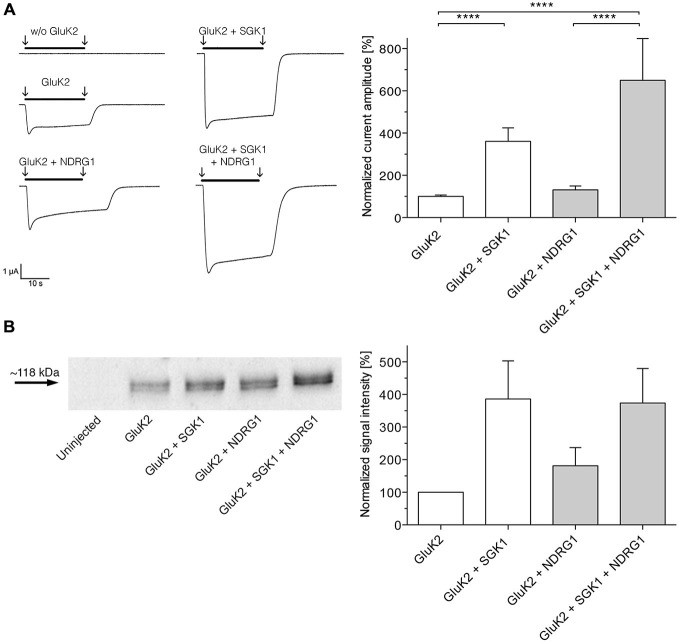
**NDRG1 does not affect SGK1-dependent modulation of GluK2 membrane expression. (A)** Representative current traces measured in *Xenopus* oocytes in response to super fusion with 300 μM glutamate. Black bars above the current traces indicate the application of glutamate (arrows indicate start and stop of application). All currents were measured at −80 mV and after pretreatment of oocytes with ConA to minimize desensitization. GluK2 current amplitudes in oocytes expressing GluK2 (*n* = 106), GluK2 + SGK1 (*n* = 85), GluK2 + NDRG2 (*n* = 19) and GluK2 + SGK1 + NDRG2 (*n* = 16) were measured and are shown normalized to GluK2 currents (bar graph). Significant differences from current amplitudes in oocytes are indicated by ****p < 0.0001. **(B)** Representative Western blot demonstrating no effect of NDRG1 on SGK1-dependent modulation of GluK2 membrane expression. Glycosylated plasma membrane proteins expressed in oocytes were labeled with biotinylated ConA. Oocytes were homogenized and plasma membrane proteins were streptavidin-precipitated. Samples including controls from uninjected oocytes were separated on an SDS gel, blotted onto a nitrocellulose membrane and probed with an anti-GluK2 antibody. The GluK2 protein has an apparent molecular mass of ~118 kDa. Bar graph showing relative abundance of GluK2 plasma membrane protein (*n* = 4). The band intensity was quantified by arithmetic analysis using the software ImageJ.

As shown in Figure 3A, coexpression of NDRG1 tended to slightly but not significantly increase the glutamate-induced current amplitude in GluK2 expressing oocytes. Coexpression of SGK1 significantly increased the glutamate-induced current amplitude in GluK2 expressing oocytes. The effect was not significantly augmented by additional coexpression of NDRG1 and correspond to the analysis of membrane expression of GluK2 in *Xenopus laevis* oocytes (Figure 3B).

### NDRG2 Phosphorylation at -T330 is Required for Suppressing the SGK1 Effect

**Figure 4:**
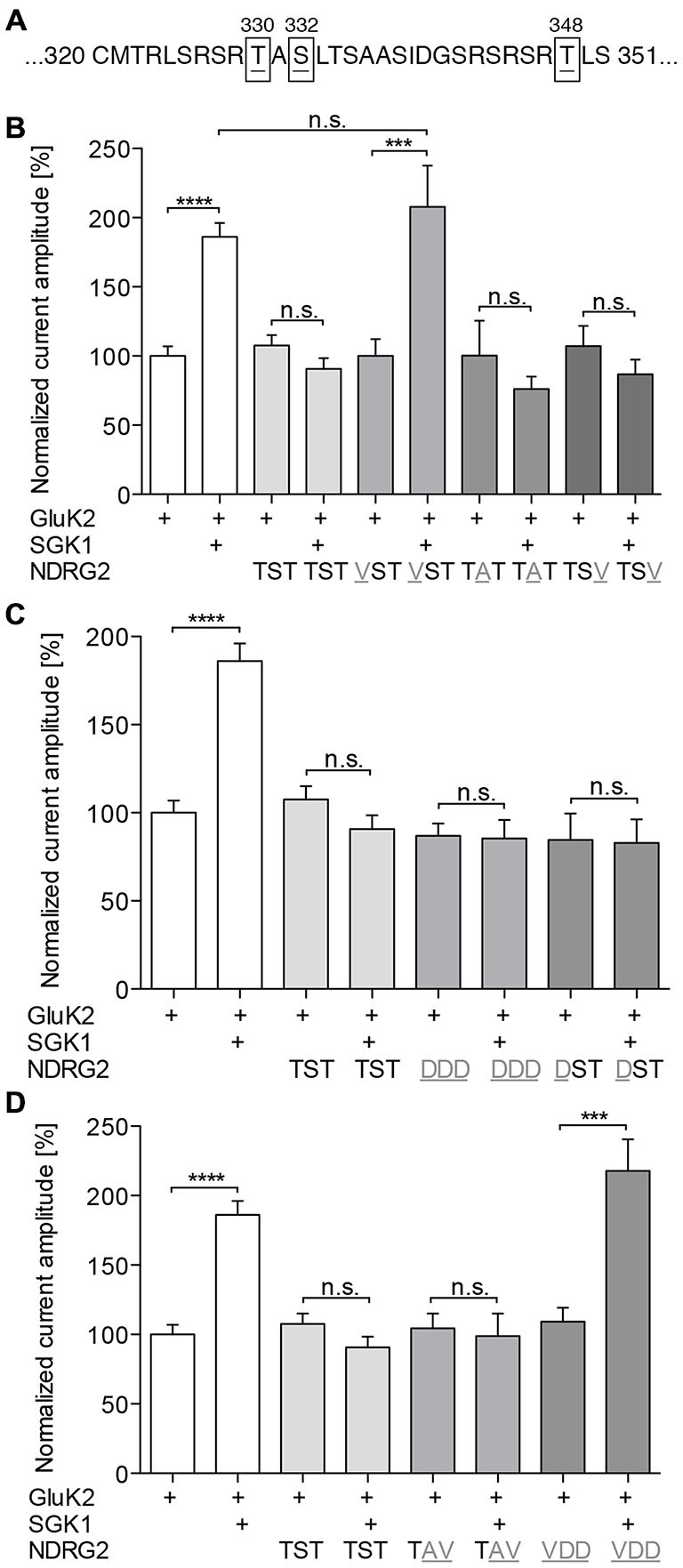
**Phosphorylation of NDRG2 is responsible for suppressing of SGK1 effect. (A)** NDRG2 amino acid sequence fragment with highlighted SGK1 phosphorylation sites T330, S332, and T348. **(B–D)** GluK2 current amplitudes in oocytes expressing GluK2 (*n* = 55), GluK2 + SGK1 (*n* = 67), and GluK2 coexpressed with NDRG2 wild type or different mutants of NDRG2 containing point mutations at SGK1 phosphorylation sites (*n* = 13–48) with and without SGK1 were measured and are shown normalized to GluK2 currents. Substitution of amino acids that are phosphorylated by SGK1 to neutral amino acids **(B)**, to phosphomimetic-amino acids **(C)** and combinations of them **(D)** were investigated. All currents were measured at −80 mV and after pretreatment of oocytes with ConA to minimize desensitization. Significant differences are indicated by ****p < 0.0001, ***p < 0.001, n.s. = not significant.

To examine the hypothesis that NDRG2 phosphorylation may be required for the suppressive effect on SGK1-dependent modulation of GluK2, we generated NDRG2 point mutants at the putative phosphorylation sites and studied the glutamate-induced current amplitudes of GluK2. Uniform expression of all NDRG2 mutants was verified in the *Xenopus* oocyte system by Western blotting (Supplementary Figure S2). First, we substituted the amino acids that are phosphorylated by SGK1 (Figure 4A), T330, S332, and T348, by neutral amino acids. The results of these substitutions are shown in Figure 4B. NDRG2^TAT^ and NDRG2^TSV^ show the same glutamate-induced current amplitudes as wild type. Coexpression of these two mutants did not significantly modulate the current amplitude in GluK2 expressing oocytes. No stimulating effect of SGK1 was observed when GluK2 was expressed together with SGK1 and NDRG2^TAT^ or NDRG2^TSV^. Expression of GluK2 with NDRG2^VST^ has no effect on GluK2, whereas a stimulating effect is observed when GluK2 is coexpressed with SGK1 and NDRG2^VST^. Further experiments were done with phosphomimetically substituted amino acids of the SGK1 phosphorylation sites on the NDRG2 protein (Figure 4C). Coexpression of GluK2 with NDRG2^DDD^ or NDRG2^DST^ showed no differences in current amplitudes compared to GluK2 alone. When SGK1 was coexpressed in both conditions, no modulatory effect of SGK1 on GluK2 currents could be observed. In addition, we performed experiments with two other NDRG2 mutants, NDRG2^TAV^ and NDRG2^VDD^. Here again, no effect could be observed on GluK2 currents upon coexpression of the NDRG2 mutants (Figure 4D). GluK2 current amplitudes were not stimulated when SGK1 was coexpressed with NDRG2^TAV^. However, coexpression of GluK2 with SGK1 and NDRG2^VDD^ showed significantly increased GluK2 current amplitudes.

### Expression of GluK2, SGK1, and NDRG2 in Rat Hippocampal Astrocytes

**Figure 5:**
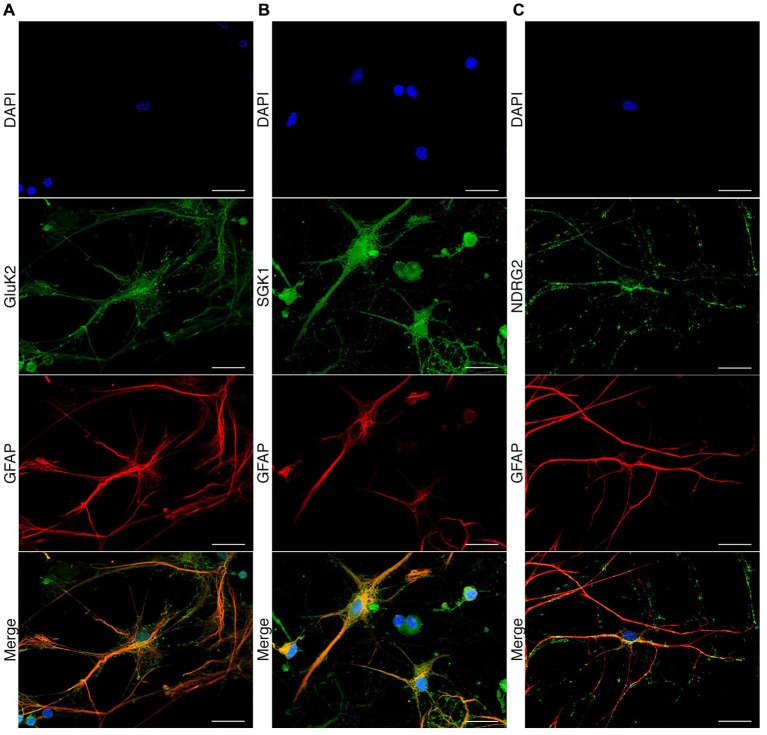
**Immunocytochemical detection of GluK2, SGK1, and NDRG2 in rat hippocampal astrocytes.** Fluorescence microscopic recordings of dissociated rat hippocampal astrocytes. Astrocytes are indicated by GFAP antigenicity (red). GluK2 (**A**, green), SGK1 (**B**, green) and NDRG2 (**C**, green) are colocalized with GFAP-labeled astrocytes. Nuclei were stained with DAPI (blue). Scale bars indicate 20 μm.

To investigate the expression of GluK2, SGK1, and NDRG2 in cultured rat hippocampal astrocytes, we performed immunocytochemical analyses using specific antibodies. Double stains for GFAP, a commonly used astrocyte marker, and GluK2 revealed colocalization of GluK2 and GFAP (Figure 5A). The expression of GluK2 in astrocytes is not limited to a specific region within the cell. Specific expression of SGK1 in astrocytes was also confirmed by double staining of SGK1 and GFAP (Figure 5B). Excessive expression of SGK1 was detected in hippocampal astrocytes. NDRG2 was detected in hippocampal astrocytes (Figure 5C), which was determined by colocalization of GFAP and NDRG2 within the astrocyte. The specificity of the commonly used GluK2 primary antibody has been shown before Hirbec et al. (2005) and Kwon et al. (2007). The specificity of NDRG2 and SGK primary antibodies was verified by Western blot using blocking peptides.

### Modulation of NDRG2 and SGK1 Protein Levels in Cultured Hippocampal Astrocytes by siRNA-Mediated Knockdown and Dexamethasone-Stimulation of Protein Expression

To investigate whether the modulation of SGK1-mediated increase of membrane expression of GluK2 described above for *Xenopus* oocytes also occurs in astrocytes we mimicked the oocyte conditions in astrocytes by modulating the protein levels of SGK1 and NDRG2.

**Figure 6:**
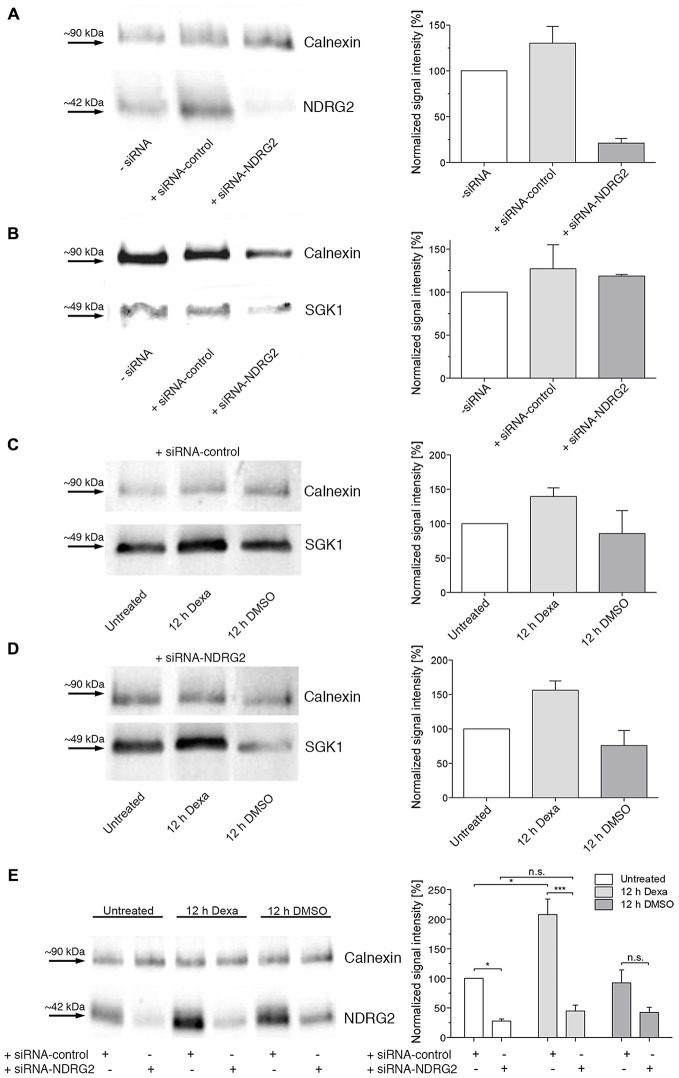
**Western blot demonstrating regulation of NDRG2 and SGK1 by siRNA and dexamethasone. (A,B)** Western blot of cytosolic NDRG2 **(A)** and SGK1 **(B)** expression levels in astrocyte enriched cultures after transfection of control siRNA or siRNA against NDRG2. As control, only transfection reagent was used (siRNA). Calnexin was used as control protein. The relative band intensities of NDRG2 and SGK1 are summarized to the right of the Western blot images (*n* = 2–3). **(C–E)** Western blot of cytosolic SGK1 **(C,D)** and NDRG2 **(E)** expression levels in astrocyte enriched cultures after transfection of control siRNA and siRNA against NDRG2 and treatment with dexamethasone for 12 h. As control, DMSO was used. Calnexin was used as control protein. The relative band intensities of SGK1 and NDRG2 are summarized to the right of the Western blot images (*n* = 2–3). Significant differences are indicated by *p < 0.05, ***p < 0.001, n.s. = not significant.

First, the NDRG2 protein level in astrocytes was down-regulated by a specific siRNA (Thermo Scientific, Schwerte, Germany). As shown in Figure 6A, the NDRG2-specific siRNA decreased the expression of NDRG2 significantly compared to cells transfected with scrambled control siRNA or mock-transfected cells. The expression level of SGK1 was not influenced by the transfection of NDRG2 siRNA and control siRNA (Figure 6B). To enhance the protein level of SGK1 in astrocytes we used the glucocorticoid dexamethasone. Dexamethasone increased the cytosolic expression of SGK1 under various conditions of transfection as presented in Figures 6C,D. Since it is known that dexamethasone has a modulatory effect on the expression level of NDRG2, we also determined the level of NDRG2 in dexamethasone-treated cells. As shown in Figure 6E, it could be verified that dexamethasone up-regulates the protein level of NDRG2 in astrocytes. NDRG2 siRNA was able to decrease the NDRG2 protein level compared to control cells in both untreated and dexamethasone-treated cells.

### Alteration of GluK2 Distribution in Astrocytes

**Figure 7:**
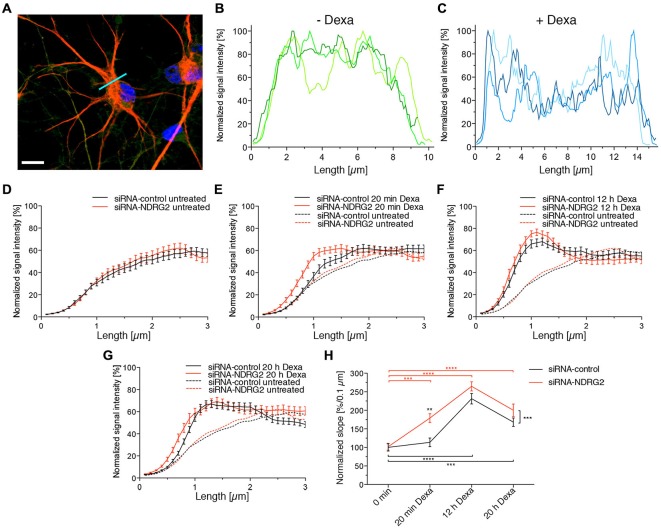
**Dexamethasone alters the GluK2 fluorescence intensity at the astrocyte membrane. (A)** Immunocytochemical staining of an astrocyte (GFAP red, DAPI blue). The light blue line identifies the location of the fluorescence profile. Scale bar = 10 μm. **(B,C)** GluK2 was immunocytochemically stained in astrocytes and fluorescence images taken with the Zeiss Observer.Z1 with ApoTome and a 63× oil immersion objective. Exemplary GluK2 fluorescence profiles of astrocytes without treatment with dexamethasone (Dexa) **(B)** and with treatment with 1 μM dexamethasone for 12 h **(C)**. **(D–G)** GluK2 fluorescence profile of the siRNA-transfected astrocytes (black: siRNA-control, red: siRNA-NDRG2) without dexamethasone treatment **(D)** and with treatment with 1 μM dexamethasone for 20 min **(E)**, 12 h **(F)**, and 20 h **(G)**. **(H)** Influence of the siRNA as well as dexamethasone treatment on the regression coefficients of the established regression line. Changes in the slope of the normalized signal intensity within the first micrometer of the transfected astrocytes when treated with 1 μM dexamethasone for different durations. GFAP and GluK2 were immunocytochemically stained in astrocytes and fluorescence images taken with the Zeiss Observer.Z1 with ApoTome and 63× oil immersion objective. Fluorescence profiles were generated in the GluK2 fluorescence channel, and normalized to the highest value of the signal intensity. Then regression lines were created within the first micrometer of the cell, their slopes were averaged and are shown in diagram **(H)**. **p < 0.01, ***p < 0.001, ****p < 0.0001, *n* = 33–48.

To determine whether the modulation of NDRG2 or SGK1 protein expression alters the membrane localization of GluK2 in astrocytes, we performed double staining for GFAP and GluK2 (Figure 7A) and determined the relative intensity of the fluorescence signal of GluK2 under different conditions (Figures 7B,C). At the beginning and the end of the cell a peak was apparent most likely reflecting the GluK2 protein at the plasma membrane. Analysis of distribution of the relative fluorescence signal was performed as described above. The slope of the regression line of the plotted fluorescence within the first micrometer of the cell was ascertained and compared among the different conditions. siRNA-transfected but untreated cells did not differ in progression of the fluorescence plot (Figure 7D). Treatment of astrocytes with dexamethasone under all conditions increased the signal intensity within the first micrometer of the cell (Figures 7E–G). At each treatment period, the signal intensity increased more strongly within siRNA NDRG2-treated cells than in scrambled siRNA control-transfected cells. As shown in Figure 7H, the values for the slope of regression lines after transfection of siRNAs but without dexamethasone treatment do not differ. Control siRNA transfected cells exhibited a significant increase in the regression coefficient after dexamethasone treatment for 12 and 20 h. Astrocytes treated with siRNA against NDRG2, showed already after 20 min of treatment with dexamethasone a highly significant increase in the regression coefficient in comparison to untreated cells. After 12 and 20 h of dexamethasone treatment, the slopes of the linear fitted lines were still significantly elevated within the first micrometer of the cells. This was not the case of the structural protein GFAP (Supplementary Figure S3). Comparison of the two groups, siRNA control and siRNA NDRG2, reveals a highly significant effect of the transfection of siRNA NDRG2 on the regression coefficients and thus the density of GluK2 at the membrane.

## Discussion

### NDRG2 Phosphorylated at T330 Suppresses the SGK1-Activated Signaling Cascade that Leads to an Increased Membrane Expression of GluK2 in a Concentration Dependent Manner

**Figure 8:**
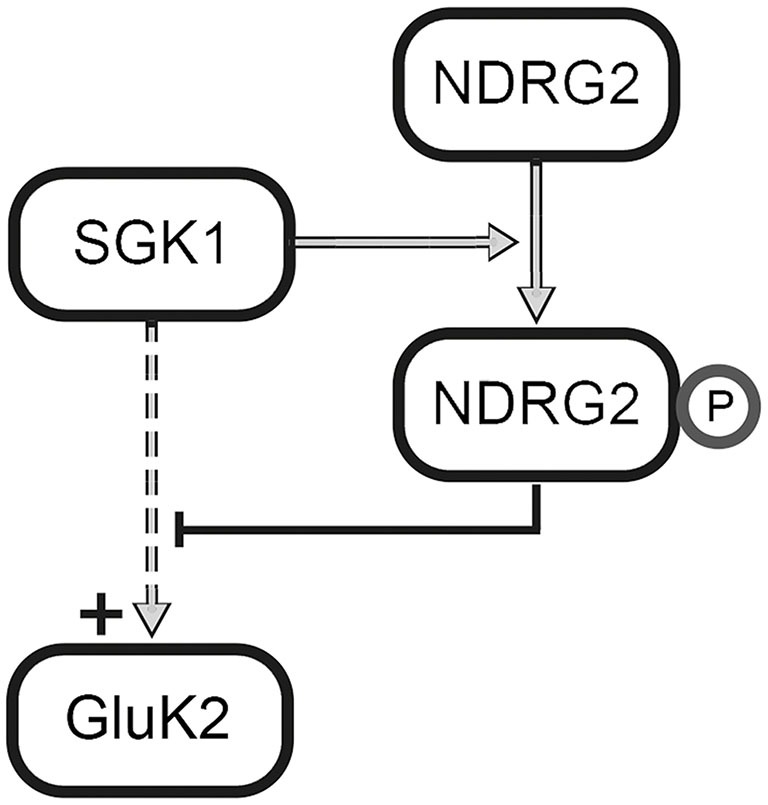
**Regulation of GluK2 by SGK1 and NDRG2.** SGK1 activates a signaling cascade that leads to the increase in membrane expression of GluK2. Further, SGK1 phosphorylates NDRG2 at phosphorylation site T330, which leads to an inhibition of the mentioned signaling cascade.

The present results reveal a novel regulator of GluK2. In Strutz-Seebohm et al. (2005) demonstrated that the serum- and glucocorticoid dependent kinase SGK1 regulates the membrane expression of GluK2. Here, we could show, that NDRG2 itself does not directly modulate expression of the kainate receptor GluK2 but suppresses the SGK1-activated increase in membrane expression of GluK2 in *Xenopus laevis* oocytes. These findings could be confirmed through Western blot analysis of membrane preparations of *Xenopus* oocytes. In addition, the study of membrane expression using EGFP-tagged fluorescent GluK2 matches with these results. Due to the absence of modulation by NDRG2 alone, a direct regulation of GluK2 by NDRG2 can be excluded. Thus, it can be concluded that NDRG2 intervenes with the SGK1-activated signaling cascade, which leads to an increase in membrane expression of GluK2. This effect of suppression of the increase in membrane expression by NDRG2 might be concentration-dependent. Upon injection of the same amount of SGK1 and NDRG2 cRNA, suppression of the SGK1 effect by NDRG2 can be observed. At a ratio of 10:1 (SGK1:NDRG2) a tendency for increase of current amplitude by SGK1 can be seen. However, a significant SGK1 effect on the GluK2 current amplitude was measured after injection of a 100-times higher amount of SGK1-cRNA compared to NDRG2-cRNA. It should be noted that no NDRG2 protein signal in Western blot could be detected when using 0.06 ng NDRG2 cRNA. It is possible that the protein amount was below the detection limit, otherwise it cannot be excludes that NDRG2 was not expressed in this condition. Nevertheless, a difference in the characteristic of the suppressive effect of NDRG2 by usage of 6 and 0.6 ng cRNA is present, which would still indicate concentration dependence. Furthermore, the suppression of increased membrane expression is dependent on the phosphorylation of NDRG2 by SGK1. Our electrophysiological measurements with different NDRG2 point mutants suggest that phosphorylation of the first SGK1 phosphorylation site of NDRG2, T330, seems to be essential for the suppressive effect on the SGK1-mediated increase of membrane expression. Mutant NDRG2 that is non-phosphorylatable at position T330 does not have the ability to intervene in the SGK1-activated signaling cascade and thus does not suppress the increase in membrane expression of GluK2 anymore. The exchange of the second or third SGK1 phosphorylation sites of NDRG2 by neutral amino acids does not alter the suppression of the SGK1 effect. The suppressive effect of T330-phosphorylated NDRG2 could also be demonstrated with NDRG2 mutants by performing a phosphomimetic substitution, i.e., inserting an aspartic acid residue at either the first (NDRG2^DST^) or at all three (NDRG2^DDD^) SGK1 phosphorylation sites. Deprotonated aspartic acid fulfills the same function as a phosphorylated tyrosine or serine. In our experiments with NDRG2^DST^, no additional effect on the current amplitude of GluK2 was observed following coexpression of SGK1. This observation suggests that the additional phosphorylation of S332 and T348 does not contribute to the suppressive effect of NDRG2. Experiments with NDRG2^TAV^ and NDRG2^VDD^ confirmed the assumption that only the first SGK1 phosphorylation site of NDRG2, T330, is essential for the suppression of increased membrane expression of GluK2. Thus, it can be concluded that only a T330-phosphorylated NDRG2 intervenes in a concentration-dependent manner in the SGK1-activated signaling pathway and prevents the increase of membrane expression of GluK2 in heterologous expression (Figure 8). In summary, phosphorylation of NDRG2 occurs parallel to the SGK1-dependent increase of GluK2 membrane expression, and phosphorylated NDRG2 suppresses the stimulation of GluK2 by SGK1.

Prior studies revealed NDRG2 as a modulator of the epithelial sodium channel ENaC (Wielpütz et al., 2007) and the Na^+^/K^+^-ATPase (Li et al., 2011). In case of ENaC, coexpression with NDRG2 leads to an increase in ENaC-mediated sodium current due to an increased membrane expression in *Xenopus* oocytes. Since ENaC is also potentiated by SGK1, experiments in which all three cRNAs were injected into oocytes were also performed (Wielpütz et al., 2007), and both the current as well as the membrane expression of ENaC were examined. In case of ENaC the mechanism of potentiation by SGK1 was already clarified (Zhou and Snyder, 2005). Briefly, SGK1 phosphorylates the ubiquitin protein ligase Nedd4-2 and thus prevents the ubiquitinylation of the channel, resulting in increased membrane expression and consequently increased current amplitude of ENaC (Zhou and Snyder, 2005; Wielpütz et al., 2007). Another mechanism of potentiation of ENaC by SGK1 is the direct phosphorylation of the epithelial Na^+^ channel (Diakov and Korbmacher, 2004). The mechanism by which NDRG2 contributes to the potentiation of ENaC remains unexplained. Since GluK2 is not directly phosphorylated by SGK1 or regulated by Nedd4-2, it is quite possible that GluK2 is regulated by another signaling cascade in which NDRG2 plays an opposite role than in the case of ENaC and thus leads to a suppression of the SGK1 effect. For example, it is known that GluK2 is directly phosphorylated by protein kinase C and then sumoylated, with the result that the endocytosis of the kainate receptor is initiated (Chamberlain et al., 2012). However, to date no involvement of NDRG2 has been reported in these processes.

### Modulation of GluK2 Membrane Expression in Primary Astrocytes of Rat Hippocampus by SGK1 and NDRG2

The expression of the three proteins of interest in astrocytes was investigated by examination of colocalization of the respective protein with GFAP in immunocytochemical staining. The staining of GFAP leads to clearly stained fibrous structures, which are characteristic for this protein. The detection of GluK2 at the protein level in astrocytes also showed distinct staining. GluK2 is localized both in the cytosol and at the membrane, consistent with previous reports (Brand-Schieber et al., 2004). Furthermore, the expression of NDRG2 in astrocytes was verified (Nichols, 2003; Okuda et al., 2008; Shen et al., 2008). Thus, the published results could be confirmed with respect to protein expression of NDRG2 in astrocytes. The expression of SGK1 in astrocytes is currently under discussion. Two reports did not detect SGK1 in astrocytes (Wärntges et al., 2002; Wu et al., 2013) whereas two other studies did (Stichel et al., 2005; Slezak et al., 2013). Here, we could prove the expression of SGK1 protein in astrocytes by showing colocalization of GFAP and SGK1 in immunocytochemical staining.

To investigate the influence of SGK1 and/or NDRG2 on GluK2 membrane expression in primary astrocytes of the hippocampus, cell culture conditions providing different levels of these proteins had to be studied. The transfection of the siRNA against NDRG2 led to a substantial reduction in the expression of this protein. Furthermore, the expression of SGK1 protein was increased by usage of dexamethasone in the cell culture medium. The observed increase in the level of SGK1 expression is consistent with experiments that have been performed in different secondary cell cultures (Webster et al., 1993; Ullrich et al., 2005). Notably, it could also be shown in Western blot experiments that dexamethasone had a stimulating effect not only on SGK1 but on the protein expression level of NDRG2 after transfection of the siRNA scrambled control, consistent with findings by Takahashi et al. (2011). Inhibition of NDRG2 expression by siRNA against NDRG2 effectively reduced the protein level of NDRG2 and abrogated the increase of NDRG2 protein abundance by dexamethasone.

### Physiological Significance of the Suppression of the SGK1-Mediated Increase in Membrane Expression of GluK2 by NDRG2 in Astrocytes

According to the present study NDRG2 suppressed the SGK1-mediated increase in membrane expression of GluK2 in astrocytes. Thus, the SGK1-mediated incorporation of the receptor in the membrane of astrocytes is reduced. NDRG2 and SGK1 sensitive regulation of GluK2 protein abundance could be of importance in various processes. For example, in cells under stress NDRG2 could prevent an inappropriately large incorporation of GluK2 receptors by engaging the SGK1-mediated signaling pathway, thereby reducing the increased membrane expression. A very high membrane expression of GluK2 in astrocytes could have a strong influence on gliotransmission and for instance, impact on the increase in the intracellular Ca^2+^ concentration following Ca^2+^ entry through the kainate receptor. Activation of GluK receptors in astrocytes may influence gene expression (McNaughton and Hunt, 1992). If NDRG2 is overexpressed, cell proliferation is inhibited (Takeichi et al., 2011; Li et al., 2013). In case of overexpression of NDRG2, the SGK1-mediated increase in membrane expression could be suppressed effectively. Thus, less GluK2 channels would be activated in the astrocyte membrane and this may have an influence on the gene expression of the cell. If NDRG2 is down-regulated at the cellular protein level, cell proliferation is stimulated (Liu et al., 2011; Takeichi et al., 2011) and more GluK2 receptors could incorporate into the astrocyte membrane and affect gene expression. Overexpression of NDRG2 in astrocytes could also have negative effects. In the case of overexpression of NDRG2, the SGK1-mediated increase in membrane expression would be suppressed and the number of GluK2 receptors would be reduced, which may compromise exocytosis of tPA. Casse et al. (2012) showed that GluK receptors in astrocytes have an important metabotropic action, resulting in a signaling cascade that inhibits the exocytosis of tPA. tPA can bind to and stimulate NMDA receptors (Nicole et al., 2001). If in a stressful situation there would be too few kainate receptors at the astrocyte membrane because of an overexpression of NDRG2, the exocytosis of tPA could not be prevented and over-excitation of neighboring neurons could occur via tPA-enhanced NMDAR function.

In summary, in this study we identify a novel, self-limiting feedback mechanism that allows for prevention of excessive SGK1-mediated GluK2 membrane expression following stress signals.

## Author Contributions

VM performed the oocyte recordings, Western Blot studies, cell culture, mutagenesis, microscopy studies, and analysis, NS-S and GS developed the concept and designed the experiments, CT performed microscopy studies, all authors edited the manuscript.

## Conflict of Interest Statement

The authors declare that the research was conducted in the absence of any commercial or financial relationships that could be construed as a potential conflict of interest.
