# Supplementary material for: NDRG2 phosphorylation provides negative feedback for SGK1-dependent regulation of a kainate receptor in astrocytes
Source: Front Cell Neurosci. 2015 Oct 6;9:387. doi: 10.3389/fncel.2015.00387 (PMC4594022; doi:10.3389/fncel.2015.00387)
Supplement: Supplementary file 1 [file Image_1.PDF]

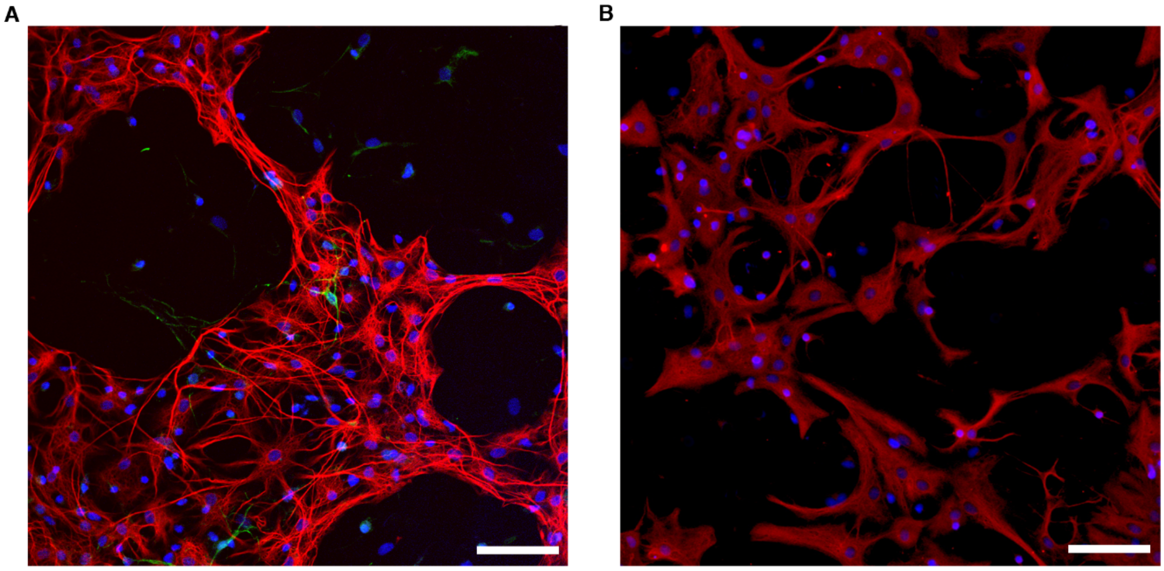

Figure S1. Immunocytochemical staining of dissociated primary rat hippocampal cultures. Fluorescence microscopic recording of dissociated rat hippocampal mixed (**A**) and astrocyte enriched cultures (**B**). Astrocytes are indicated by GFAP antigenicity (red) and neurons by Neurofilament M (green). Nuclei were stained with DAPI (blue). Scale bar indicate 50 µm.
