# Supplementary material for: NDRG2 phosphorylation provides negative feedback for SGK1-dependent regulation of a kainate receptor in astrocytes
Source: Front Cell Neurosci. 2015 Oct 6;9:387. doi: 10.3389/fncel.2015.00387 (PMC4594022; doi:10.3389/fncel.2015.00387)
Supplement: Supplementary file 2 [file Image_2.PDF]

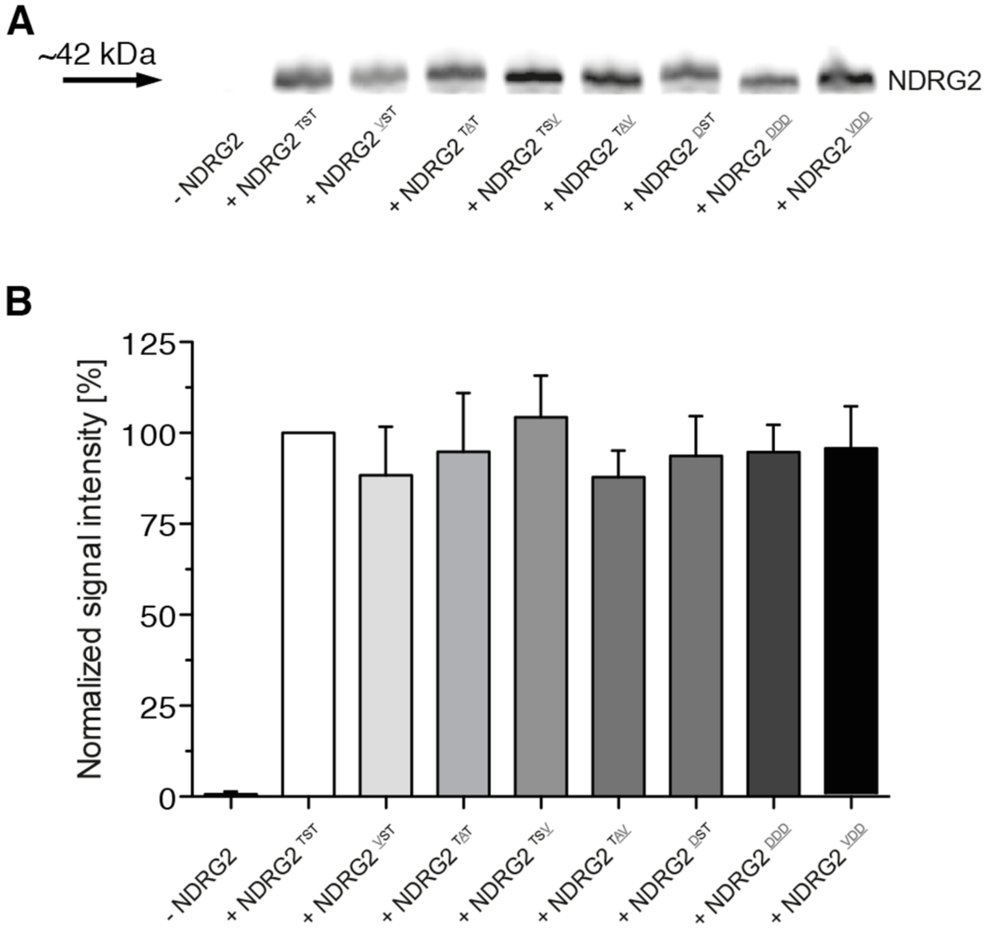

Figure S2. Uniform expression of all NDRG2 mutants. (**A**) Exemplary Western blot of cytosolic expressed NDRG2-varients after injection of the corresponding cRNAs and an expression time of 5-6 days. Oocytes were homogenized. Samples including controls from uninjected oocytes (-NDRG2) were separated on an SDS gel, blotted onto a nitrocellulose membrane and probed with an anti-NDRG2 antibody. The NDRG2 protein has an apparent molecular mass of ~42 kDa. (**B**) Quantification of Western blots performed to detect the cytosolic expressed NDRG2 mutants using ImageJ. The respective signal intensities were normalized to the value of the "+ $NDRG2^{TST}$". The introduction of point mutations into NDRG2-gene does not change the expression of the protein in *Xenopus laevis*-oocytes (n = 4).
