# Supplementary material for: NDRG2 phosphorylation provides negative feedback for SGK1-dependent regulation of a kainate receptor in astrocytes
Source: Front Cell Neurosci. 2015 Oct 6;9:387. doi: 10.3389/fncel.2015.00387 (PMC4594022; doi:10.3389/fncel.2015.00387)
Supplement: Supplementary file 3 [file Image_3.PDF]

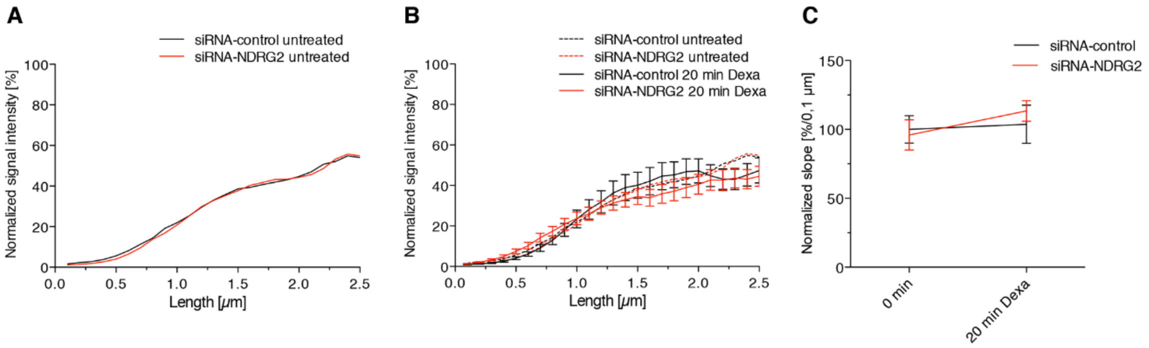

Figure S3. Dexamethason has no effect on GFAP fluorescence intensity at the astrocyte membrane. GFAP was immunocytochemically stained in astrocytes and fluorescence images taken with the Zeiss Observer.Z1 with ApoTome and a 63x oil immersion objective. (**A**) GFAP fluorescence profile of the siRNA-transfected astrocytes (black: siRNA-control, red: siRNA-NDRG2) without dexamethasone treatment. (**B**) GFAP fluorescence profile of the siRNA-transfected astrocytes (black: siRNA-control, red: siRNA-NDRG2) without dexamethasone treatment (dotted lines) and with treatment with 1 µM dexamethasone (solid line) for 20 min. (**C**) Influence of the siRNA as well as dexamethasone treatment on the regression coefficients of the established regression line. Changes in the slope of the normalized signal intensity within the first micrometer of the transfected astrocytes when treated with 1 µM dexamethasone for 20 min. GFAP was immunocytochemically stained in astrocytes and fluorescence images taken with the Zeiss Observer.Z1 with ApoTome and 63x oil immersion objective. Fluorescence profiles were generated in the GFAP fluorescence channel, and normalized to the highest value of the signal intensity. Then regression lines were created within the first micrometer of the cell, their slopes were averaged and are shown in diagram **C**. The treatment of the cultures with siRNA or dexamethasone did not alter the fluorescence intensity at the astrocyte membrane. n = 18-23.
